# Supplementary figures and images for: vB_CacS-HV1 as a Novel Pahexavirus Bacteriophage with Lytic and Anti-Biofilm Potential against Cutibacterium acnes
Source: Microorganisms. 2024 Jul 31;12(8):1566. doi: 10.3390/microorganisms12081566 (PMC11356600; doi:10.3390/microorganisms12081566)

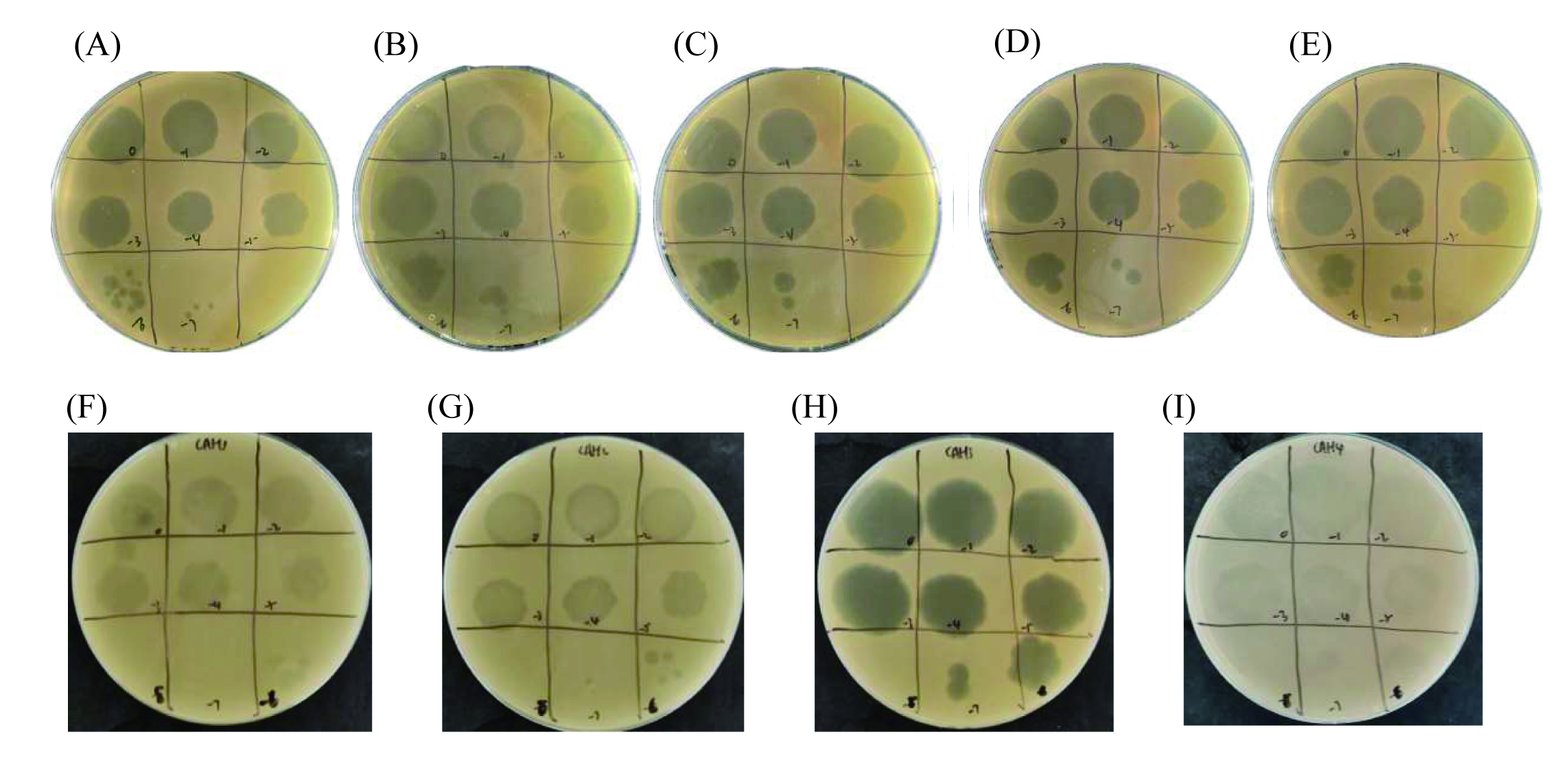

Supplement: Supplementary file 1 [file microorganisms-12-01566-s001.zip › Figure S1 Plaque morphology of phage vB_CacS-HV1 on susceptible host strains.tif]
